# Kinetic Chain Rehabilitation: A Theoretical Framework

**DOI:** 10.1155/2012/853037

**Published:** 2012-05-14

**Authors:** Aaron Sciascia, Robin Cromwell

**Affiliations:** Lexington Clinic, Shoulder Center of Kentucky, 1221 South Broadway, Lexington, KY 40504, USA

## Abstract

Sequenced physiologic muscle activations in the upper and lower extremity result in an integrated biomechanical task. This sequencing is known as the kinetic chain, and, in upper extremity dominant tasks, the energy development and output follows a proximal to distal sequencing. Impairment of one or more kinetic chain links can create dysfunctional biomechanical output leading to pain and/or injury. When deficits exist in the preceding links, they can negatively affect the shoulder. Rehabilitation of shoulder injuries should involve evaluation for and restoration of all kinetic chain deficits that may hinder kinetic chain function. Rehabilitation programs focused on eliminating kinetic chain deficits, and soreness should follow a proximal to distal rationale where lower extremity impairments are addressed in addition to the upper extremity impairments. A logical progression focusing on flexibility, strength, proprioception, and endurance with kinetic chain influence is recommended.

## 1. Introduction

Dynamic upper extremity dominant tasks such as throwing, hitting, and serving occur as the result of the integrated, multisegmented, sequential joint motion, and muscle activation system known as the kinetic chain. Proper utilization of the kinetic chain allows maximal force to be developed in the core which can then be efficiently transferred to the arm during these actions. In order for the tasks to be effective and efficient, the kinetic chain links (the different body segments) must have optimal amounts of muscle flexibility, strength, proprioception, and endurance as well as the ability to perform the task consistently on a repetitive basis. Proper kinetic chain sequences referred to as biomechanical “nodes” have been previously described for baseball pitchers and tennis players [1, 2]. When these nodes are not achieved, increased load and stress may occur on the shoulder and elbow joints which can lead to pain or injury. The focus for clinicians is to identify the cause(s) which led or contributed to the impairment. The clinician must then implement injury rehabilitation and prevention programs which will initially eliminate physical deficits followed by a focus on increasing an athlete's longevity while simultaneously decreasing the risk of injury. The purpose of this paper is to present a theoretical framework which focuses on maximizing kinetic chain utilization and output, accomplished through improving flexibility of all involved joints and soft tissue, strengthening the lower extremity and core musculature, optimizing scapular control, and improving muscular endurance of persons experiencing shoulder pain. 

## 2. Rationale and Stages of Rehabilitation

The kinetic chain rehabilitation approach is not unlike other treatment philosophies in that the early or acute stage of rehabilitation is focused on protecting healing tissue and reducing pain. This is traditionally accomplished with protection (rest and/or immobilization), anti-inflammatory medication, and selected therapeutic modalities. However, these remedies are designed to treat the symptoms rather than the cause of dysfunction, therefore, a clinician must not place extraneous amounts of effort in this phase or consider these treatments as the core of the therapy program.

Following initial protection, the patient should be transitioned into what is known as the recovery phase of rehabilitation [3]. At this point, a logical, progressive plan of treatment is implemented where muscle reeducation and soft tissue mobility become the focal points with respect to the stages of tissue healing in early rehabilitation. Since the core drives kinetic chain function, it is imperative that optimal stabilization and force generation can occur. Muscle reeducation of the core muscles should begin early and target both local and global muscles [4]. In this stage of the kinetic chain approach, soft tissue deficits, that is inflexibilities of both upper and lower extremities, should be addressed because if left unattended, these deficits can impede progressions into the later stages of the treatment process.

Following the correction of the surrounding deficits, the next step in the logical progression would be to direct the treatment efforts on stabilizing the scapula. Primary scapular stabilization and motion on the thorax involves coupling of the upper and lower fibers of the trapezius muscle with the serratus anterior and rhomboid muscles. The lower trapezius has a role as a scapular stabilizer when the arm is lowered from an elevated position by helping maintain the scapula against the thorax [5]. The serratus anterior contributes to all components of three-dimensional motion of the scapula during arm elevation helping to produce scapular upward rotation, posterior tilt, and external rotation while stabilizing the medial border and inferior angle preventing scapular winging [6].

The scapular position that allows optimal muscle activation of the shoulder joint muscles to occur is that of retraction and external rotation which results from synergistic muscle activations in patterns from the hip and trunk through the scapula to the arm, which then facilitates maximal muscle activation of the muscles attached to the scapula [3]. This integrated sequencing allows the retracted scapula to serve as a stable base for the origin of all the rotator cuff muscles, allowing optimal concavity compression to occur [7]. Therefore, implementing scapular stabilization exercises which incorporate lower extremity stability and muscle activation would be appropriate.

At this latter point in the rehabilitation process (the functional phase), general glenohumeral strengthening would be introduced. Open chain exercises attempt to isolate the rotator cuff muscles through long lever, single plane ranges of motion which could potentially create shear across the joint creating muscular irritation. The exercises are often performed in nonfunctional positions (prone or supine) which discourages proper kinetic chain activation [8–10]. Only after the kinetic chain links have been optimized should traditional strengthening measures be introduced however; the measures should also be tailored to involve the kinetic chain links as an integrated unit rather than in isolation in order to properly encourage and simulate normal function.

## 3. Guidelines for Eliminating Dysfunction(Rehabilitation)

The factors contributing to dysfunction of the arm in overhead athletics can be traced to anatomical and biomechanical causes both locally and distally to the site of symptoms. Shoulder pain can result from bony pathology such as acromioclavicular or sternoclavicular injury, fractures to the clavicle or humerus, and bone spur/osteophyte formation. It can also derive from soft tissue causes such as labral injury, rotator cuff disease, or glenohumeral instability. These injured or altered structures may require surgical repair in order for rehabilitation to be successful. Pain may also occur as a result of altered mechanics/kinematics which can occur as the result of muscle weakness and/or tightness in one or more muscle groups in either the upper or lower extremity.

In the event the anatomical tissue has not been compromised, clinical focus should be on reestablishing optimal segmental activation in order to redevelop arm function. Functional tasks are dependent upon appropriate functioning of the kinetic chain as a unit, optimization of the individual components (proper flexibility and strength), and appropriate coordination of the individual segments. Each segment plays a critical role in helping an individual achieve optimal athletic performance. For example, the large muscles of the lower extremity are designed to generate power and create a firm stable base of support. This stable base allows core muscles to activate causing the trunk to have dynamic stability so the arm can direct the resultant energy in the overhead throwing motion. In the event that one or more of the segments fail to properly generate or transfer energy along the kinetic chain, the load distribution and force output become altered making the task being performed less efficient and effective. Over time, this decreased efficacy can cause otherwise healthy tissue to become irritated and stressed leading to injury.

The ideal principles for integrated functional kinetic chain rehabilitation which help assure optimal functioning of each segment are: to (1) establish proper postural alignment, (2) establish proper motion at all involved segments, (3) facilitate scapular motion via exaggeration of lower extremity/trunk movement, (4) exaggerate of scapular retraction in controlling excessive protraction, (5) utilize the closed chain exercise early, and (6) work in multiple planes.

### 3.1. Consider Postural Influences

The common proximal (in relation to the ground) causes of distal dysfunction include poor rear foot control, a lack of ankle range of motion, hip extensor and abductor tightness and/or weakness, limited spinal mobility, limited pelvic motion/strength, and poor scapular control. These unaddressed deficits lead to dysfunction along the kinetic chain resulting in poor rehabilitation outcomes.

The local and global stabilizers of the trunk together provide optimal core stability. The larger global muscles including the abdominal muscles and erector spinae, and hip abductors are vital to power generation and stability for upper extremity function. The incorporation of core strengthening into rehabilitation regimens has been shown to increase hip extensor muscle strength [11] resulting in pain reduction and an increase of the overall strength of the pelvis and trunk postural muscles of patients with low back pain [12]. In order to create a stable base, the rehabilitation protocols should focus on the primary stabilizing musculature such as the transverse abdominus and multifidi which are responsible for segmental spinal stability and alignment. The internal/external obliques, erector spinae, rectus abdominus, and the quadratus lumborum should then be incorporated for trunk stability. This stage of rehabilitation should not be overlooked. The core, being the most proximal component of the kinetic chain (in relation to the arm), is the critical link between the development of and transfer of energy.

### 3.2. Establish Proper Motion

Most postural concerns can be addressed by improving the flexibility of the musculature and/or the mobility of the bony components. Flexibility of both the upper and lower extremity can be increased via standard static, dynamic, and/or ballistic stretching. Based on previous findings regarding flexibility deficits in upper extremity dominant athletes, the hamstring, hip flexor, hip adductors, hip rotator, and gastrocnemius/soleus muscle groups should be targeted for the lower extremity. Improving lower extremity muscle flexibility has been linked to improving lower body movement patterns and improving overall athletic performance [13–17]. The pectoralis minor, latissimus dorsi, and posterior shoulder muscles should be the point of focus for the upper extremity [18–22].

**Figure 1:**
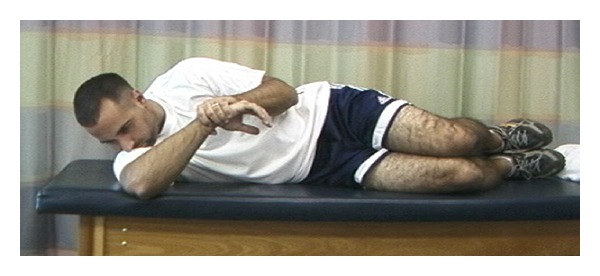
Sleeper stretch.

**Figure 2:**
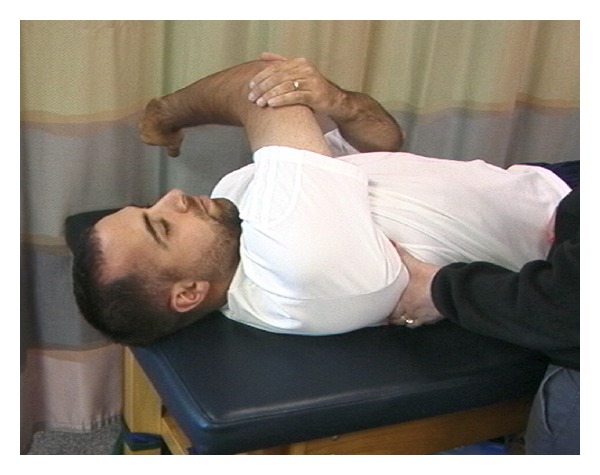
Cross-body stretch.

**Figure 3:**
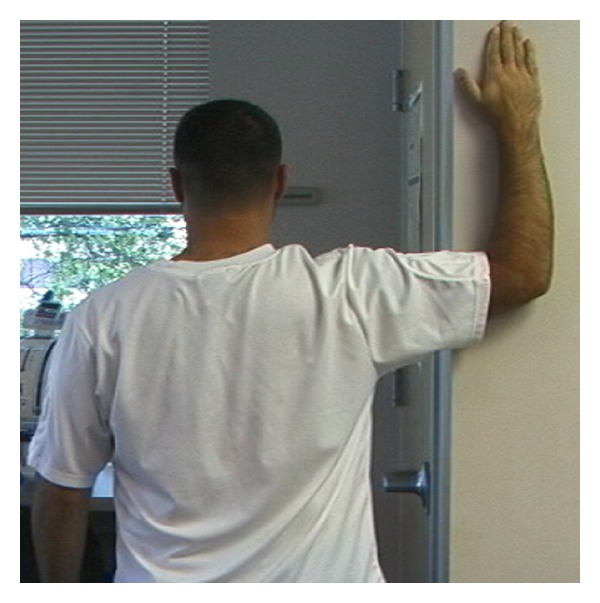
Corner stretch.

Specifically in overhead athletes, it has been shown that acute and chronic changes in muscle due to eccentric load can affect the amount of overall shoulder motion [23, 24]. These findings in overhead athletes have led to the recommendation of incorporating stretching into the treatment regimen. To address the adaptive posterior shoulder tightness [25–27], GIRD, and anterior shoulder tightness [28], utilization of the cross body stretch [25, 26, 29–31] (Figure 1), sleeper stretch [29, 32] (Figure 2), and corner stretching [33] (Figure 3) have been found to be effective.

### 3.3. Facilitate Scapular Motion

**Figure 4:**
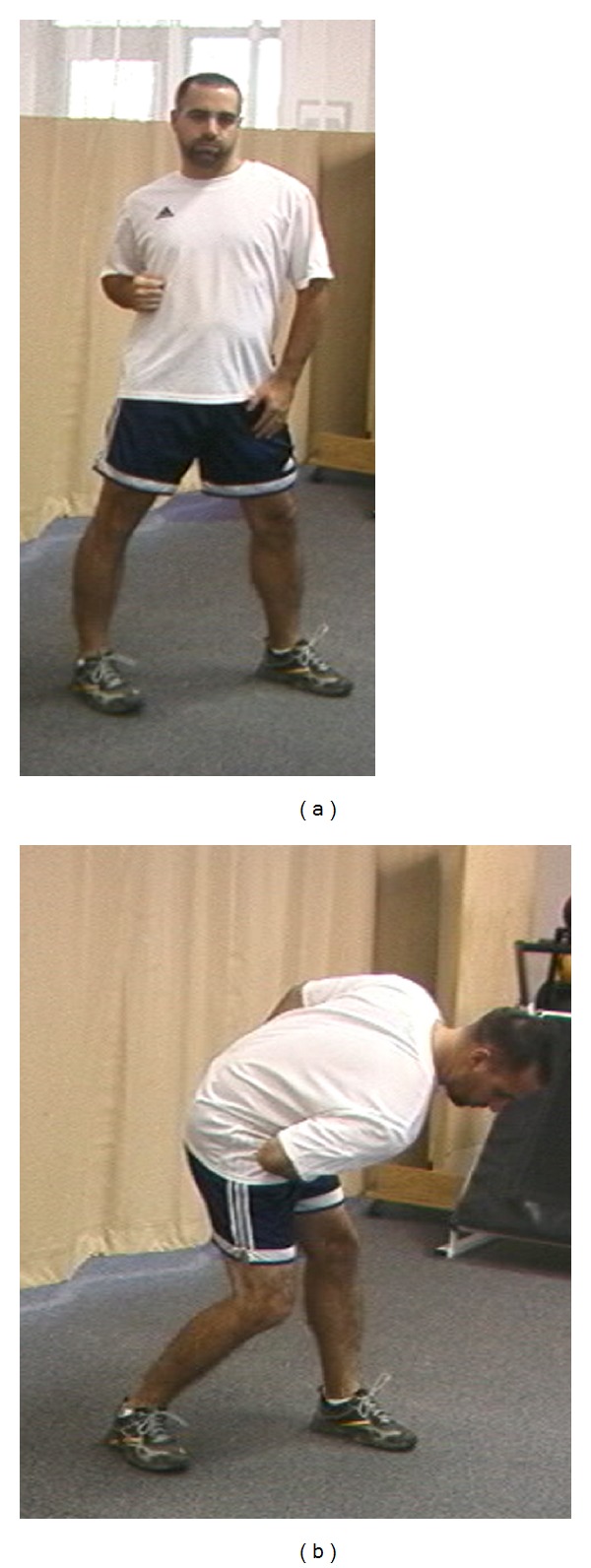
(a) Facilitation of scapular retraction: hip and trunk extension facilitates scapular retraction. (b) Facilitation of scapular protraction: hip and trunk flexion facilitate scapular protraction.

Periscapular muscles such as the serratus anterior and lower trapezius should be a point of focus in early training and rehabilitation. Early training should incorporate the trunk and hip in order to facilitate proximal to distal sequencing of muscle activation. It is important to remember that scapular rotation is accessory in nature whereas scapular translation is physiologic or voluntary. Therefore, implementing exercises which attempt to isolate scapular rotation are not functional and should be discouraged. Utilizing the lower extremity in order to encourage scapular motion is ideal in that it mimics kinetic chain sequencing. Minimal stress is placed on the glenohumeral joint during hip and trunk extension which facilitate scapular retraction. All exercises are started with the feet on the ground and involve hip extension and pelvic control. The patterns of activation are both ipsilateral and contralateral. Diagonal motions involving trunk rotation around a stable leg simulate the normal pattern of throwing (Figures 4(a) and 4(b)). As the shoulder heals and is ready for motion and loading in the intermediate or recovery stage of rehabilitation, the patterns can include arm movement as the final part of the exercise. Therefore, specific closed chain exercises known as the low row and inferior glide, which have been shown to activate the serratus and lower trapezius at safe levels of muscle activation, need to be incorporated into a facilitatory kinetic chain rehabilitation program [34].

Exploitation of the transverse plane helps accentuate both scapular retraction and protraction. By forcing proximal stability, the hip and trunk muscle activations, which have been demonstrated to precede arm motion, will be more effective during a specified task [35]. In addition to generating and transferring energy to the distal segments, this component of rehabilitation allows the utilization of the stable base for arm motion [36]. Rehabilitation programs should attempt to encourage stimulation of proper proprioceptive feedback as well, so the patient can return to their desired level of function [3, 37].

### 3.4. Scapular Retraction for Protraction Control

Scapular protraction is a necessary kinematic translation which occurs during the ball release through follow-through phases of the throwing motion. Protraction occurs, via serratus anterior activation, during the throwing motion as a primary mechanism in maintaining contact between the humeral head and glenoid fossa. Protraction also occurs during the deceleration phase of throwing as the arm moves forward [38, 39].

**Figure 5:**
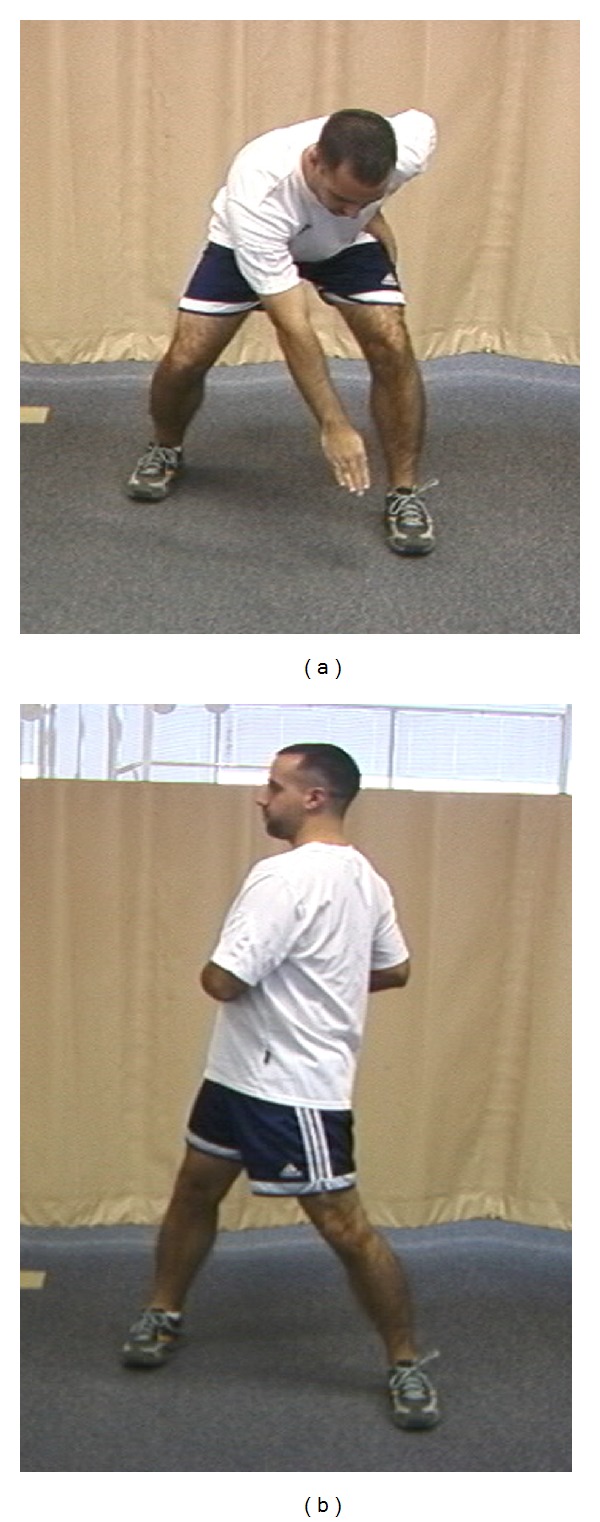
(a) Starting position for the lawnmower maneuver. (b) This exercise accentuates scapular external rotation through the use of the transverse plane.

The serratus anterior muscle is a multifunctional muscle designed to move and stabilize the scapula in various positions of arm elevation. One of the muscle's more important functions is to externally rotate the scapula which occurs at terminal scapular retraction (Figures 5(a) and 5(b)). This is the scapular position that allows optimal muscle activation of the shoulder joint muscles to occur. Scapular retraction is an obligatory and integral part of normal scapulohumeral rhythm in coupled shoulder motions and functions. It results from synergistic muscle activations in patterns from the hip and trunk through the scapula to the arm, which then facilitates maximal muscle activation of the muscles attached to the scapula. The retracted scapula then can act as a stable base for the origin of all the rotator cuff muscles so they can activate optimally.

Excessive scapular protraction does not allow optimal rotator cuff activation to occur [40–42]. It has been found that demonstrated rotator cuff strength increased as much as 24% when the scapula was stabilized and retracted [40, 43]. The muscles responsible for performing scapular retraction can help control scapular protraction through eccentric control. When optimized, these muscles can properly maintain scapular stability thus decreasing excessive protraction with arm movement. For this reason, the early phases of training should focus on scapular strengthening in an attempt to restore normal scapular kinematics rather than placing an early emphasis on rotator cuff strengthening as performed in more traditional rehabilitation protocols.

### 3.5. Early Closed Chain Implementation

Kinetic chain-based rehabilitation activities have been grouped into open and closed chain [44]. Typically, when soft tissue pathologic, closed chain exercises are implemented early in the rehabilitation process. There are 3 components which make usage of closed kinetic chain exercise advantageous in early rehabilitation. First, the exercise environment can be controlled. This allows the focus to be taken away from the arm as an integrated unit with high dynamic demands and place it in a stable, axially loaded, and static setting. Second, closed chain exercise is ideal for working “at” specific ranges of motion compared to working “through” a range of motion which helps provide a “snapshot” within the full arc of normal motion. Finally, closed chain exercise allows the rotator cuff and scapular musculature to be unloaded by decreasing the amount of force generated and stress applied to the involved soft tissue. These types of exercises are best suited for reestablishing the proximal stability and control in the links of the kinetic chain such as the pelvis and trunk. Open chain exercises, which generate greater loads in comparison to closed chain activities, should be utilized later in rehabilitation programs due to their increased demand on the soft tissue due to the longer arm levers these exercises require.

**Figure 6:**
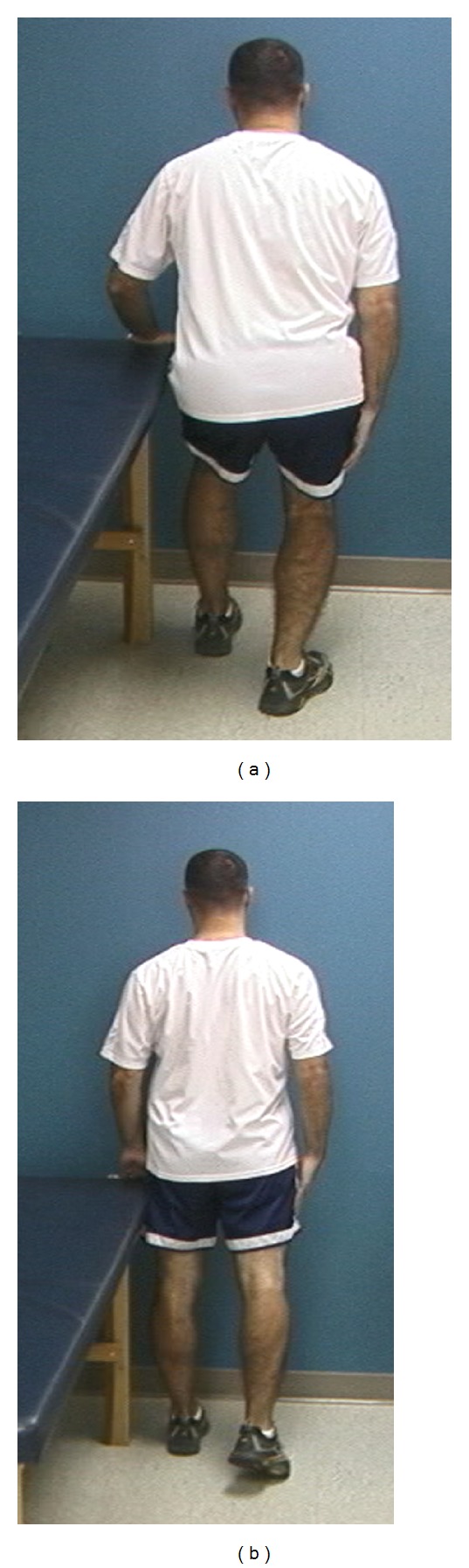
(a) Starting position for low row exercise. (b) Terminal position for low row exercise.

The rationale behind the closed-chain framework is to maximize the ability of the inhibited muscles to activate. This involves placing the extremity in a closed-chain position, emphasizing normal activation patterns, and focusing on the muscle of interest by deemphasizing compensatory muscle activation. For example, if a patient presents with shrugging during arm elevation, then it can be assumed that the lower trapezius and/or serratus anterior are not working effectively enough during the dynamic task. A closed chain exercise such as the low row should be utilized because the short lever positioning in conjunction with the pelvis and trunk acting as the driver facilitates lower trapezius and serratus anterior coactivation which decrease the activation of the upper trapezius [34] (Figures 6(a) and 6(b)). This is the normal muscle activation pattern for scapular retraction and depression. Once the normal activation pattern has been restored, then more challenging isolated exercises can be employed.

### 3.6. Work in Multiple Planes

Strengthening and stabilization should begin by emphasizing work in successful planes and then progress to deficient planes. Clinicians should avoid the use of single planar exercises which isolate specific muscles or specific joints. Greater isolation should be utilized in the later stages of the rehabilitation protocol. During the early phases, emphasis should be placed on achieving successful positions, motions, and muscle activation sequences. In this manner, normal physiologic activations are restored, which lead to restoration of normal biomechanical motions.

Most activities, whether they are sports-related or normal daily movements, occur in the transverse plane. Therefore, the transverse plane should be exploited particularly in the early phases of rehabilitation. The protocol should progress to more unilateral planes as normal scapulohumeral kinematics are restored.

### 3.7. Maintenance Programs

**Figure 7:**
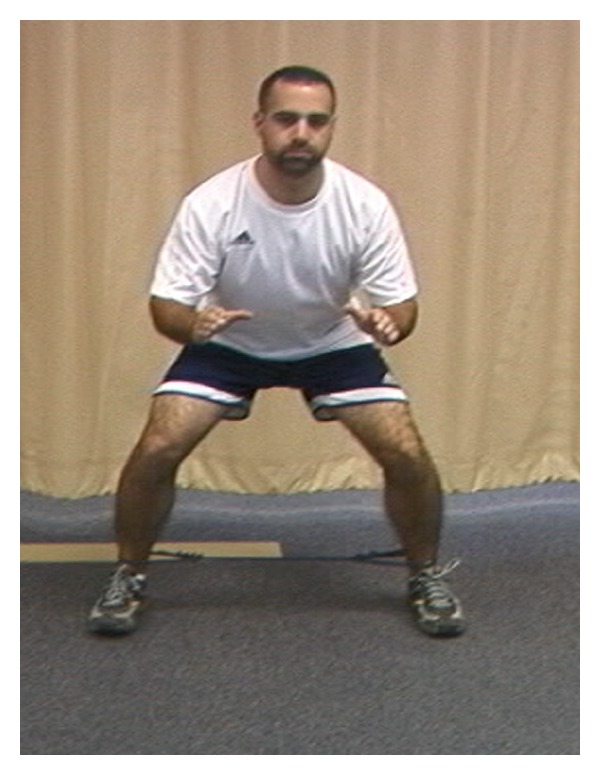
Side stepping.

**Figure 8:**
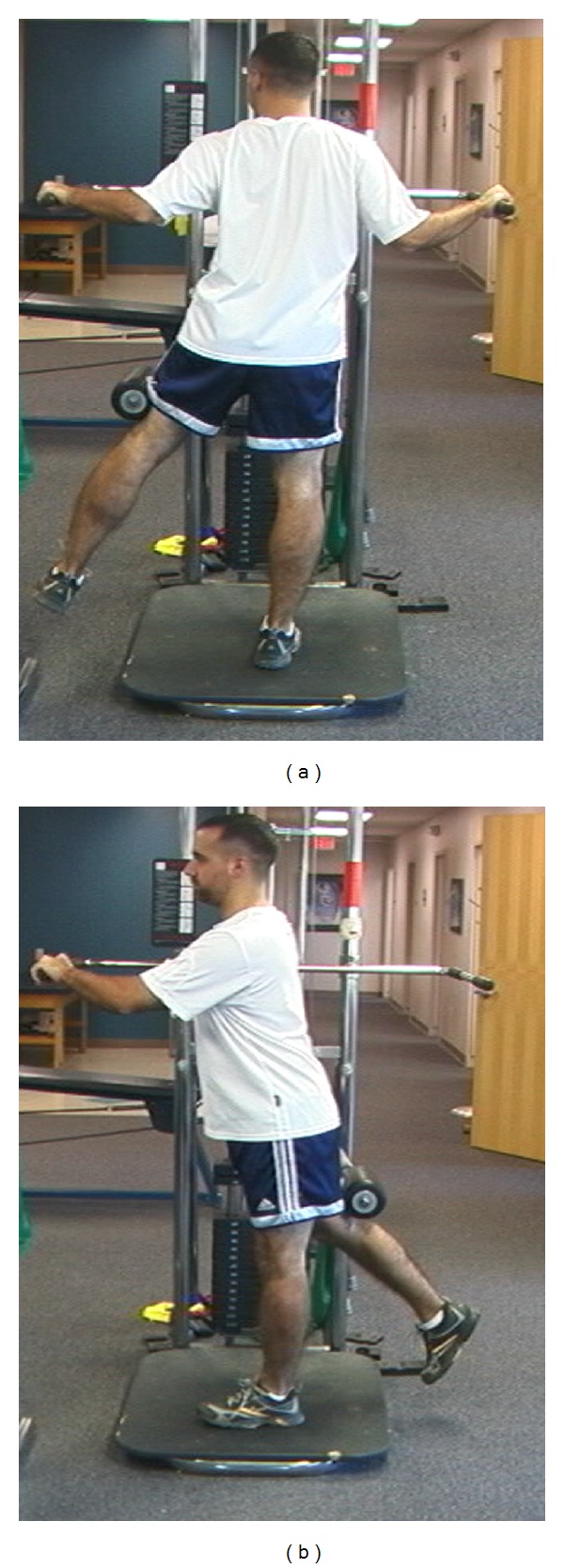
(a) Examples of hip abduction strengthening. (b) Examples of hip extension strengthening.

**Figure 9:**
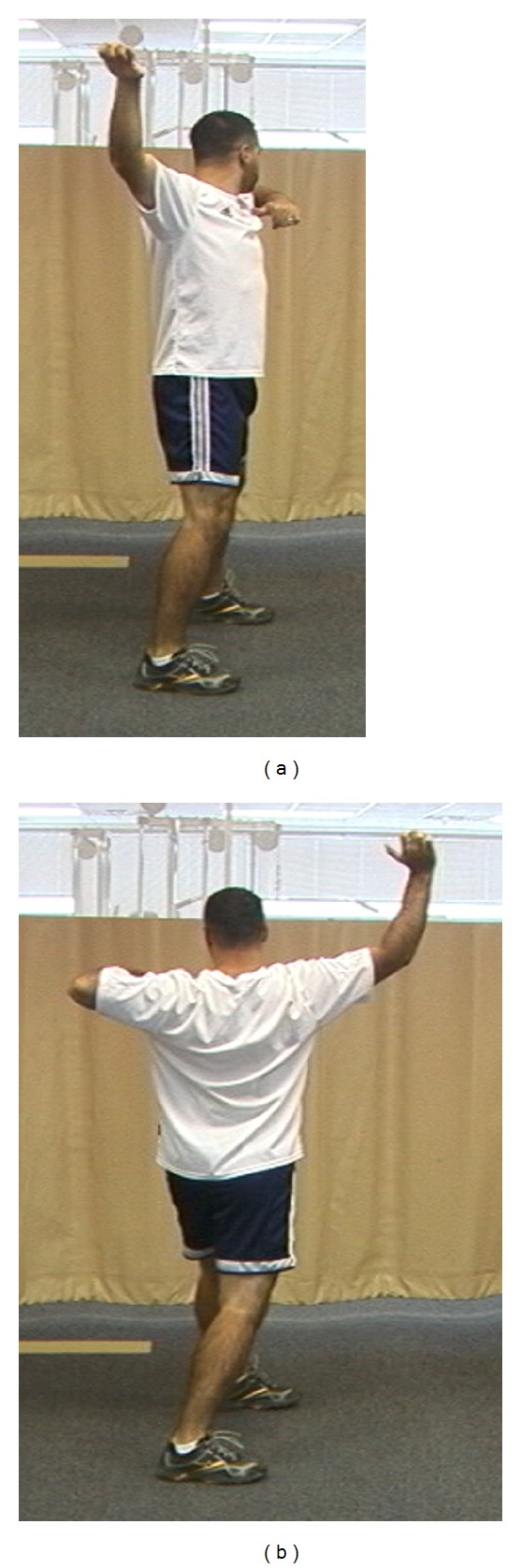
(a) Power position begins with the dominant arm in the 90/90 position and forearm pronated. (b) Next, while maintaining the 90/90 arm position, rotate the trunk forward to simulate the throwing motion phases of acceleration to ball release.

**Figure 10:**
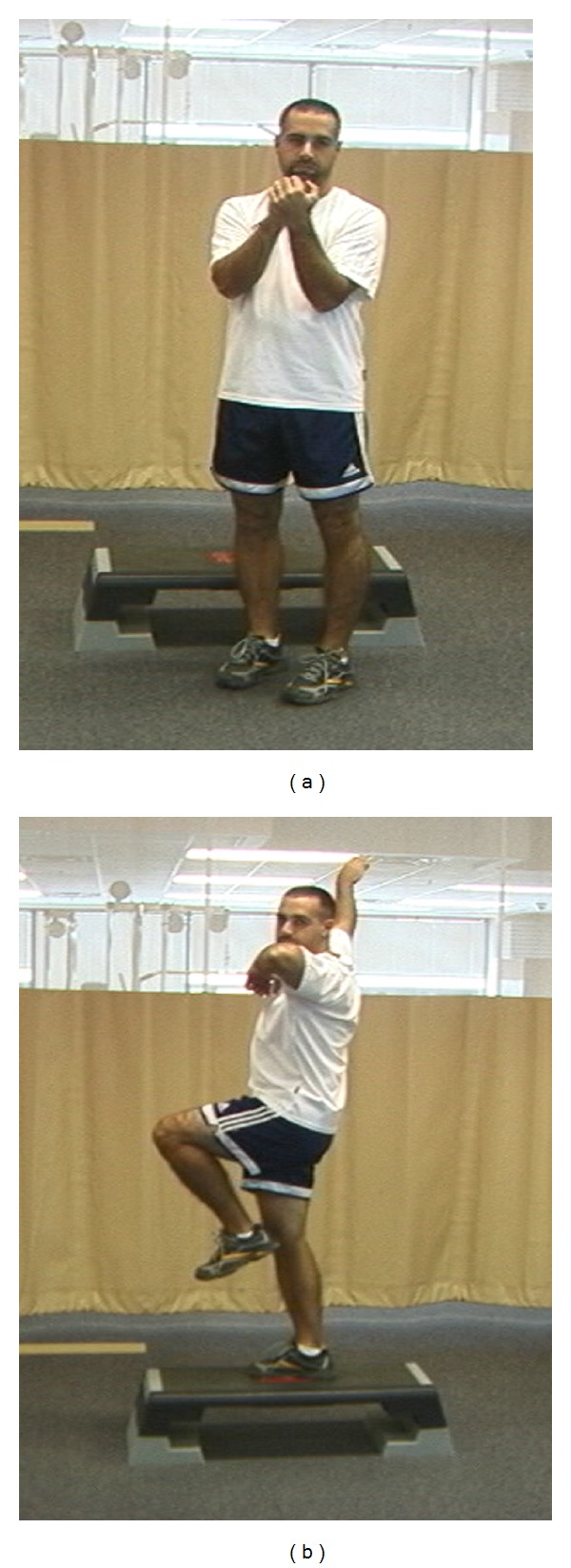
(a) Starting position for the step back exercise. (b) Step back with power position encouraging use of a stable back leg.

**Figure 11:**
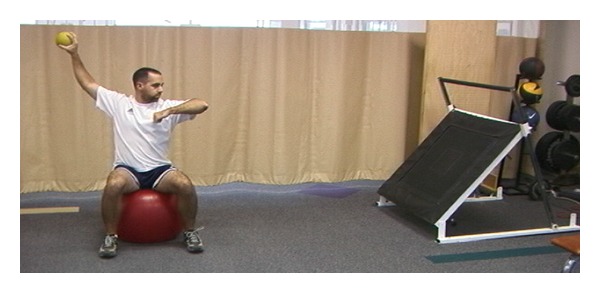
Rebounder with power position.

Once the kinetic chain deficits have been corrected, and normal kinematics have been restored, the focus should transition to muscle endurance and proprioception. Three areas of focus should be implemented: lower extremity muscle power and endurance, integrated sports-specific exercise, and upper extremity power and endurance. High repetition exercises designed to increase lower extremity muscle endurance should be employed first. For example, pitching is a task requiring activation of multiple segments; repetitively, adequate muscle endurance of all involved muscle groups is necessary for optimal performance. Focus on the gastrocnemius/soleus, quadriceps, hamstrings, and hip abductor muscle groups would be recommended (Figures 7, 8(a), and 8(b)). The next component would be the utilization of integrated sports-specific exercise which encourages use of the improved lower extremity muscle strength and endurance to help facilitate upper extremity muscle activation. This is accomplished through synchronous single leg and transverse plane exercises which aid in improving proprioception as well as muscle education (Figures 9(a), 9(b), 10(a), and 10(b)). The final area, upper extremity power and endurance, is addressed via high repetition, long lever exercises performed in standing and prone positions (Figure 11).

General arm pain not generated by disrupted anatomy or kinetic chain deficit suggests that the extremity is being used too often or incorrectly. Excessive use or repetition without appropriate recovery time leads to muscular fatigue which in turn decreases muscular activity and force production, subsequently causing biomechanical abnormalities (decreased cocking, dropped elbow), all of which can result in pain or soreness. Adequate rest and recovery should be allotted in order for muscular function to be less affected by the stress of physical activity.

### 3.8. Return to Play

Following the alleviation of pain and soreness, restoration of the kinetic chain deficits, and improvement in strength and endurance of the necessary muscles, throwing progressions can be applied [45, 46]. Ideally, the following items need to be accounted for determining return to play: (1) optimal kinetic chain links (pelvis control over planted leg, effective hip and trunk extension), (2) scapular retraction achievement while controlling scapular protraction, (3) proper flexibility of upper and lower extremity, and (4) advancement through functional throwing progression without regression of prior deficits/symptoms.

## 4. Summary

Rehabilitation of the throwing athlete's shoulder should follow a kinetic chain-based regimen that addresses specific deficits within individual links which can aid in restoring the natural proximal to distal muscle activation sequencing. The deficits can be addressed through a logical progression of therapeutic interventions focusing on flexibility, strength, proprioception, and endurance with integrated kinetic chain components. Preventative or prospective exercises to minimize future loading stresses should be included at the end of rehabilitation as part of the return to function.

## References

[1] Lintner D, Noonan TJ, Kibler WB (2008). Injury patterns and biomechanics of the athlete's shoulder. *Clinics in Sports Medicine*.

[2] Davis JT, Limpisvasti O, Fluhme D (2009). The effect of pitching biomechanics on the upper extremity in youth and adolescent baseball pitchers. *American Journal of Sports Medicine*.

[3] Kibler WB, McMullen J, Uhl T (2000). Shoulder rehabilitation strategies, guidelines, and practice. *Operative Techniques in Sports Medicine*.

[4] Kibler WB, Press J, Sciascia A (2006). The role of core stability in athletic function.. *Sports Medicine*.

[5] Bagg SD, Forrest WJ (1988). A biomechanical analysis of scapular rotation during arm abduction in the scapular plane. *American Journal of Physical Medicine and Rehabilitation*.

[6] Ludewig PM, Cook TM, Nawoczenski DA (1996). Three-dimensional scapular orientation and muscle activity at selected positions of humeral elevation. *Journal of Orthopaedic and Sports Physical Therapy*.

[7] Lippitt S, Vanderhooft JE, Harris SL, Sidles JA, Harryman DT, Matsen FA (1993). Glenohumeral stability from concavity-compression: a quantitative analysis. *Journal of Shoulder and Elbow Surgery*.

[8] Blackburn TA, McLeod WD, White B, Wofford L (1990). EMG analysis of posterior rotator cuff exercises. *Journal of Athletic Training*.

[9] Townsend H, Jobe FW, Pink M, Perry J (1991). Electromyographic analysis of the glenohumeral muscles during a baseball rehabilitation program. *American Journal of Sports Medicine*.

[10] Ballantyne BT, O’Hare SJ, Paschall JL (1993). Electromyographic activity of selected shoulder muscles in commonly used therapeutic exercises. *Physical Therapy*.

[11] Nadler SF, Malanga GA, Bartoli LA, Feinberg JH, Prybicien M, Deprince M (2002). Hip muscle imbalance and low back pain in athletes: influence of core strengthening. *Medicine and Science in Sports and Exercise*.

[12] Petrofsky JS, Batt J, Brown J (2008). Improving the outcomes after back injury by a core muscle strengthening program. *Journal of Applied Research*.

[13] Ross MD (2007). Effect of a 15-day pragmatic hamstring stretching program on hamstring flexibility and single hop for distance test performance. *Research in Sports Medicine*.

[14] Bandy WD, Irion JM, Briggler M (1998). The effect of static stretch and dynamic range of motion training on the flexibility of the hamstring muscles. *Journal of Orthopaedic and Sports Physical Therapy*.

[15] Higgs F, Winter SL (2009). The Effect of a four-week proprioceptive neuromuscular facilitation stretching program on isokinetic torque production. *Journal of Strength and Conditioning Research*.

[16] Worrell TW, Smith TL, Winegardner J (1994). Effect of hamstring stretching on hamstring muscle performance. *Journal of Orthopaedic and Sports Physical Therapy*.

[17] Yuktasir B, Kaya F (2009). Investigation into the long-term effects of static and PNF stretching exercises on range of motion and jump performance. *Journal of Bodywork and Movement Therapies*.

[18] Lephart SM, Smoliga JM, Myers JB, Sell TC, Tsai YS (2007). An eight-week golf-specific exercise program improves physical characteristics, swing mechanics, and golf performance in recreational golfers. *Journal of Strength and Conditioning Research*.

[19] Tsai YS, Sell TC, Smoliga JM, Myers JB, Learman KE, Lephart SM (2010). A Comparison of physical characteristics and swing mechanics between golfers with and without a history of low back pain. *Journal of Orthopaedic and Sports Physical Therapy*.

[20] Vad VB, Bhat AL, Basrai D, Gebeh A, Aspergren DD, Andrews JR (2004). Low back pain in professional golfers: the role of associated hip and low back range-of-motion deficits. *American Journal of Sports Medicine*.

[21] Tyler TF, Nicholas SJ, Roy T, Gleim GW (2000). Quantification of posterior capsule tightness and motion loss in patients with shoulder impingement. *American Journal of Sports Medicine*.

[22] Robb AJ, Fleisig G, Wilk K, MacRina L, Bolt B, Pajaczkowski J (2010). Passive ranges of motion of the hips and their relationship with pitching biomechanics and ball velocity in professional baseball pitchers. *American Journal of Sports Medicine*.

[23] Reinold MM, Wilk KE, Macrina LC (2008). Changes in shoulder and elbow passive range of motion after pitching in professional baseball players. *American Journal of Sports Medicine*.

[24] Freehill MT, Ebel BG, Archer KR (2011). Glenohumeral range of motion in major league pitchers: Changes over the playing season. *Sports Health*.

[25] Tyler TF, Nicholas SJ, Lee SJ, Mullaney M, McHugh MP (2010). Correction of posterior shoulder tightness is associated with symptom resolution in patients with internal impingement. *American Journal of Sports Medicine*.

[26] Sauers E, August A, Snyder A (2007). Fauls stretching routine produces acute gains in throwing shoulder mobility in collegiate baseball players. *Journal of Sport Rehabilitation*.

[27] Myers JB, Laudner KG, Pasquale MR, Bradley JP, Lephart SM (2006). Glenohumeral range of motion deficits and posterior shoulder tightness in throwers with pathologic internal impingement. *American Journal of Sports Medicine*.

[28] Borstad JD, Ludewig PM (2005). The effect of long versus short pectoralis minor resting length on scapular kinematics in healthy individuals. *Journal of Orthopaedic and Sports Physical Therapy*.

[29] Burkhart SS, Morgan CD, Kibler WB (2003). The disabled throwing shoulder: spectrum of pathology part III: the SICK scapula, scapular dyskinesis, the kinetic chain, and rehabilitation. *Arthroscopy*.

[30] Sciascia A, Kibler WB (2006). The pediatric overhead athlete: what is the real problem?. *Clinical Journal of Sport Medicine*.

[31] McClure P, Balaicuis J, Heiland D, Broersma ME, Thorndike CK, Wood A (2007). A randomized controlled comparison of stretching procedures for posterior shoulder tightness. *Journal of Orthopaedic and Sports Physical Therapy*.

[32] Laudner KG, Sipes RC, Wilson JT (2008). The acute effects of sleeper stretches on shoulder range of motion. *Journal of Athletic Training*.

[33] Borstad JD, Ludewig PM (2006). Comparison of three stretches for the pectoralis minor muscle. *Journal of Shoulder and Elbow Surgery*.

[34] Ben Kibler W, Sciascia AD, Uhl TL, Tambay N, Cunningham T (2008). Electromyographic analysis of specific exercises for scapular control in early phases of shoulder rehabilitation. *American Journal of Sports Medicine*.

[35] Zattara M, Bouisset S (1988). Posturo-kinetic organisation during the early phase of voluntary upper limb movement. I. Normal subjects. *Journal of Neurology Neurosurgery and Psychiatry*.

[36] Cordo PJ, Nashner LM (1982). Properties of postural adjustments associated with rapid arm movements. *Journal of Neurophysiology*.

[37] Lephart SM, Henry TJ (1996). The physiological basis for open and closed kinetic chain rehabilitation for the upper extremity. *Journal of Sport Rehabilitation*.

[38] Perry J, Rowe CR (1988). Muscle control of the shoulder. *The Shoulder*.

[39] Kibler WB (1998). The role of the scapula in athletic shoulder function. *American Journal of Sports Medicine*.

[40] Kibler WB, Sciascia A, Dome D (2006). Evaluation of apparent and absolute supraspinatus strength in patients with shoulder injury using the scapular retraction test. *American Journal of Sports Medicine*.

[41] Smith J, Kotajarvi BR, Padgett DJ, Eischen JJ (2002). Effect of scapular protraction and retraction on isometric shoulder elevation strength. *Archives of Physical Medicine and Rehabilitation*.

[42] Smith J, Dietrich CT, Kotajarvi BR, Kaufman KR (2006). The effect of scapular protraction on isometric shoulder rotation strength in normal subjects. *Journal of Shoulder and Elbow Surgery*.

[43] Tate AR, McClure P, Kareha S, Irwin D (2008). Effect of the scapula reposition test on shoulder impingement symptoms and elevation strength in overhead athletes. *Journal of Orthopaedic and Sports Physical Therapy*.

[44] Kibler WB, Livingston B (2001). Closed-chain rehabilitation for upper and lower extremities.. *The Journal of the American Academy of Orthopaedic Surgeons*.

[45] Wilk KE, Meister K, Andrews JR (2002). Current concepts in the rehabilitation of the overhead throwing athlete. *American Journal of Sports Medicine*.

[46] Axe MJ, Windley TC, Snyder-Mackler L (2001). Data-based interval throwing programs for baseball position players from age 13 to college level. *Journal of Sport Rehabilitation*.

